# Executive function and high ambiguity perceptual discrimination contribute to individual differences in mnemonic discrimination in older adults

**DOI:** 10.1016/j.cognition.2020.104556

**Published:** 2021-04

**Authors:** Helena M. Gellersen, Alexandra N. Trelle, Richard N. Henson, Jon S. Simons

**Affiliations:** a Department of Psychology, University of Cambridge, Cambridge, UK; b Department of Psychology, Stanford University, Palo Alto, USA; c MRC Cognition and Brain Sciences Unit, and Department of Psychiatry, University of Cambridge, UK

**Keywords:** Aging, Memory, Mnemonic discrimination, Perceptual discrimination, Executive function, Individual differences

## Abstract

Mnemonic discrimination deficits, or impaired ability to discriminate between similar events in memory, is a hallmark of cognitive aging, characterised by a stark age-related increase in false recognition. While individual differences in mnemonic discrimination have gained attention due to potential relevance for early detection of Alzheimer's disease, our understanding of the component processes that contribute to variability in task performance across older adults remains limited. The present investigation explores the roles of representational quality, indexed by perceptual discrimination of objects and scenes with overlapping features, and strategic retrieval ability, indexed by standardised tests of executive function, to mnemonic discrimination in a large cohort of older adults (*N*=124). We took an individual differences approach and characterised the contributions of these factors to performance under Forced Choice (FC) and Yes/No (YN) recognition memory formats, which place different demands on strategic retrieval. Performance in both test formats declined with age. Accounting for age, individual differences in FC memory performance were best explained by perceptual discrimination score, whereas YN memory performance was best explained by executive functions. A linear mixed model and dominance analyses confirmed the relatively greater importance of perceptual discrimination over executive functioning for FC performance, while the opposite was true for YN. These findings highlight parallels between perceptual and mnemonic discrimination in aging, the importance of considering demands on executive functions in the context of mnemonic discrimination, and the relevance of test format for modulating the impact of these factors on performance in older adults.

## Highlights

• Executive functions and perceptual discrimination impact mnemonic discrimination in aging.
• The relative contribution of these factors depends on demands placed on strategic retrieval.
• In a Forced Choice task, perceptual discrimination explains individual differences in memory.
• In a Yes/No task, executive functions best explain individual variability in performance.
• Studies should consider choice of task format when aiming to isolate specific mnemonic processes.

## 1 Introduction

Aging is associated with impairments in mnemonic discrimination, or the ability to distinguish between events in memory that share overlapping features (Stark, Yassa, Lacy, & Stark, 2013). Specifically, older adults are more likely than younger adults to incorrectly endorse novel items as previously studied when they are perceptually similar to studied items (i.e., ‘lures’) but not when they are distinct (i.e. novel lures; Stark & Stark, 2017; Stark, Stevenson, Wu, Rutledge, & Stark, 2015; Trelle, Henson, Green, & Simons, 2017). Age-related differences in mnemonic discrimination have gained considerable interest, and this pattern of impairment has been well-documented by many different groups (Pidgeon & Morcom, 2014; Reagh et al., 2016; Stark et al., 2015; Stark & Stark, 2017; Trelle et al., 2017). In large part, this interest has been due to potential utility of mnemonic discrimination tasks for detecting early changes in the brain associated with preclinical Alzheimer's disease (Berron et al., 2019; Maass et al., 2019; Rentz et al., 2013; Stark et al., 2013; Stark, Kirwan, & Stark, 2019; Trelle et al., in press; Webb, Foster, Horn, Kennedy, & Rodrigue, 2020). This idea emerged from observations that performance relies critically on the hippocampus and adjacent entorhinal and perirhinal cortex (Reagh et al., 2018; Reagh & Yassa, 2014), brain areas that are among the earliest affected by neurofibrillary tangle pathology (Braak & Braak, 1991). Despite growing interest in individual differences in mnemonic discrimination performance, which may be clinically meaningful, our understanding of the component processes that contribute to variability in performance across older adults remains limited. Here we focus on two candidate factors: the ability to form detailed representations of items to allow for differentiation of highly similar targets and lures, and the ability to strategically retrieve these details (Trelle et al., 2017). We further contrast their relative contributions to individual differences in mnemonic discrimination as a function of test format.

Existing work exploring individual differences in mnemonic discrimination in cognitively unimpaired older adults has primarily described the impact of hippocampal integrity on performance (Bennett, Stark, & Stark, 2019; Stark et al., 2019) and has shown that delayed recall tests are related to mnemonic discrimination performance (Migo et al., 2014; Toner, Pirogovsky, Kirwan, & Gilbert, 2009; Trelle et al., 2017). While this work has provided important initial insights into factors that contribute to individual differences in mnemonic discrimination in older adults, few studies have examined factors beyond hippocampal-dependent processes (e.g., pattern separation and pattern completion) that may give rise to elevated rates of lure false recognition with age.

MTL does not act in isolation to support mnemonic processes. The prefrontal cortex (PFC) supervises the MTL system during memory encoding and retrieval (Simons & Spiers, 2003), supporting goal-directed attention, selection, and inhibition processes, as well as the maintenance and evaluation of retrieved features in working memory (Badre & Wagner, 2007; Blumenfeld & Ranganath, 2007). PFC-mediated executive functions (EF) are affected by age and increases in false recognition have been linked to age-related changes in prefrontal cortex function (Devitt & Schacter, 2016; Guerin, Robbins, Gilmore, & Schacter, 2012; McDonough, Wong, & Gallo, 2013; Nyberg, 2017; Schacter & Slotnick, 2004; Straube, 2012). For instance, older adults are impaired in engaging in ‘recall-to-reject’ (Migo, Montaldi, Norman, Quamme, & Mayes, 2009; Trelle et al., 2017), a cognitively demanding retrieval strategy that plays a key role in minimising false recognition by recalling specific item features of the study episode that can be compared to those of similar lures in order to reject them as novel (Cohn, Emrich, & Moscovitch, 2008; Gallo, 2004; Gallo, Bell, Beier, & Schacter, 2006). Thus, individual differences in cognitive control of memory necessary to support recall-to-reject may also impact mnemonic discrimination ability. However, prior work investigating the role of executive functions on mnemonic discrimination is mixed, with some studies failing to identify effects (Davidson, Vidjen, Trincao-Batra, & Collin, 2019; Toner et al., 2009) and others suggesting executive functions may play a role when the test format posed demands on strategic retrieval (Foster & Giovanello, 2020; Trelle et al., 2017). Notably, however, these studies had relatively small samples (20–40 older adults) and focused on group comparisons. The degree to which individual differences in executive functions impact mnemonic discrimination in older adults therefore remains unclear.

Importantly, age-related increases in false recognition are also observed in experimental conditions in which demands on controlled retrieval processes are minimized (Trelle et al., 2017; Yeung, Ryan, Cowell, & Barense, 2013), and age-dependent impairments in object discrimination even exist outside the realm of episodic memory tasks, such as in perceptual oddity tasks (Burke et al., 2018; Burke, Ryan, & Barnes, 2012; Newsome, Duarte, & Barense, 2012; Ryan et al., 2012). As in memory studies, age-related impairment in perceptual discrimination tasks are observed specifically under conditions in which targets and lures share overlapping features (i.e., conditions of high feature ambiguity), but not when they are distinct, and therefore can be distinguished on the basis of simple features. Notably, prior evidence from neuroimaging, patients with MTL lesions, and non-human primates indicates that perceptual discrimination under conditions of high feature ambiguity relies critically on regions within the MTL (Barense, Henson, Lee, & Graham, 2010; Bussey and Saksida, 2002, Bussey and Saksida, 2005; Kent, Hvoslef-Eide, Saksida, & Bussey, 2016; Lee et al., 2005; Lee, Yeung, & Barense, 2012; Murray & Bussey, 1999). Collectively, this work suggests that common representations may support both perceptual and mnemonic tasks that involve resolving interference between stimuli with overlapping features, and that aging may reduce the availability of these representations (Barense et al., 2012; Bussey & Saksida, 2005; Kent et al., 2016; Newsome et al., 2012). Thus, performance on perceptual discrimination (PD) tasks may provide an index of representational quality, or individual differences in the ability to form representations that can disambiguate stimuli with overlapping features. However, work exploring the relationship between complex perceptual discrimination and mnemonic discrimination is extremely limited (Trelle et al., 2017), and no studies to date have examined continuous relationships between these factors in a large sample of older adults.

Although multiple factors may contribute to false recognition with age, their relative contribution to performance may differ depending on how memory is tested. Specifically, multiple test formats have been used to assess false recognition of similar lures. The first, and most commonly adopted, is the format in which a single probe is presented, either a target, a similar lure or an unrelated foil, and individuals judge its study history. In the paradigm presented in our study we refer to this test format as ‘Yes/No’ (YN), where participants answer to the question whether the probe was previously seen (‘yes’) or novel (‘no’). Alternatively, memory performance can also be assessed using a Forced Choice (FC) test, in which targets and corresponding lures are presented simultaneously.

Critically, prior work indicates that rates of false recognition across older and younger adults are reduced considerably in the FC format (Guerin et al., 2012; Migo et al., 2009; Trelle et al., 2017). Existing evidence suggests that this is due, in part, to reduced demands on the hippocampus (Holdstock et al., 2002; Norman, 2010) and PFC-dependent recall-based retrieval strategies (e.g., recall-to-reject) when targets and corresponding lures are presented together (Migo et al., 2009; Trelle et al., 2017). Consistent with this possibility, prior work in older adults has demonstrated that the relationship between neuropsychological tests of recall and recognition differs as a function of test format (Migo et al., 2014). Specifically, whereas recall explained variance in performance when targets and lures were presented individually, measures of recognition predicted performance when targets and corresponding lures were presented side by side. Taken together, this work suggests that test format impacts the accessibility of stimulus representations, perhaps by modulating demands on strategic retrieval processes (Trelle et al., 2017).

In the present study, we examine the relative contributions of individual differences in high ambiguity perceptual discrimination as index of representational quality, and executive function as index of the ability to engage strategic retrieval processes, to mnemonic discrimination performance in a large cohort of older adults. We further assess whether the contribution of each factor varies as a function of test format. We assayed the integrity of complex perceptual representations using perceptual oddity tasks that involve disambiguating objects and scenes with overlapping features, which have been used in patient and neuroimaging work exploring MTL contributions to perception (Barense et al., 2010; Lee et al., 2005). In accordance with prior studies on cognitive aging, executive functions were indexed using traditional neuropsychological tests, including those measuring working memory, task switching, inhibition, and semantic fluency (Davidson & Glisky, 2002; Glisky, Rubin, & Davidson, 2001; Hedden et al., 2012; Trelle et al., 2017).

We first sought to replicate the previously reported age-group differences in memory performance across task formats (Trelle et al., 2017) and in perceptual discrimination accuracy under conditions of high feature ambiguity (Ryan et al., 2012) by comparing older adults' performance to a group of healthy younger adults. Second, we sought to characterise relationships between perceptual discrimination, executive function, and memory performance in each test format among older adults, controlling for effects of age. We predicted that perceptual discrimination performance would be sufficient to explain Forced Choice performance, whereas Yes/No performance would be primarily influenced by individual differences in executive function, due to greater demands on strategic retrieval processes in the Yes/No task. We significantly add to prior work by simultaneously considering the influence of perception and executive function on individual differences in mnemonic discrimination.

## 2 Methods

### 2.1 Participants

Fifty-two younger adults aged 18 to 35 (*M*=23.0, *SD*=3.85; *N*=32 female) and 124 cognitively unimpaired older community-dwelling adults aged 60 to 87 (*M*=70.3, *SD*=5.39; *N*=75 female) participated in this study. Participants were native English speakers, had normal or corrected to normal vision, and no history of diagnosed psychiatric or neurological conditions. Volunteers provided written informed consent for participation in a manner approved by the Cambridge Psychology Research Ethics Committee. All volunteers were reimbursed for their time.

All older adults performed within the normal range (≥26) on the Montreal Cognitive Assessment (MoCA) screening tool for cognitive impairment (Damian et al., 2011; Nasreddine et al., 2005). We used the Memory Functioning Questionnaire (MFQ) to obtain a measure of subjective memory complaints among older adults (Gilewski, Zelinski, & Schaie, 1990). We focused on subscales that describe the frequency (32 items) and seriousness of forgetting (18 items) on a 7-point Likert scale, where 1 corresponded to serious problems and 7 to no problems at all. Older adults reported only moderate cases of forgetting in terms of frequency and seriousness (see Table 1 for details). Thus, no participants were excluded on the basis of objective or subjective cognitive impairment, and the present sample was deemed cognitively unimpaired.

**Table 1:** Sample demographics.

	**Younger adults**	**Older adults**
*N* (female)	52 (32)	124 (75)
Age	23.02 (3.85)	70.35 (5.39)
Education	16.83 (3.13)	17.33 (4.86)
MoCA	NA	28.26 (1.32)
MFQ forgetting	NA	5.12 (0.70)
MFQ seriousness	NA	4.56 (1.31)
Shipley	33.56 (4.50)	37.50 (1.78)
NART	34.88 (6.77)	42.22 (5.04)

*Note*: Means and standard deviations indicated in parentheses. *MFQ forgetting*=score on the Memory Functioning Questionnaire Frequency of Forgetting Subscale, averaged for a number of different categories (e.g. appointments, personal dates, names, etc.), where scores range from 1="always presents a problem" to 7="never presents a problem". *MFQ seriousness*=MFQ Seriousness of Forgetting Subscale, where instances of forgetting are rated in terms of their seriousness, ranging from 1="very serious" to 7="not serious"; *MoCA*=Montreal Cognitive Assessment (maximum 30; normal cognition ≥26). NART=National Adult Reading Test. Shipley=Vocabulary test, Shipley Institute of Living Scale.

Older and younger adults did not differ in terms of years of education (*t*<1, *p*>.4). However, older adults had higher crystallised IQ as measured using the Vocabulary test of the Shipley Institute of Living Scale (Shipley, 1986; *t*(35.87)=−5.00, *p*<.001, *d*=1.51) and the National Adult Reading Test (NART; Nelson & Willison, 1991; *t*(156)=−6.95, *p*<.001, *d*=1.35). Note that only a subsample of younger adults completed the full battery of neuropsychological tests (N=34).

We did not select the sample size based on a priori power calculation, but rather collected the maximal sample size that was feasible given our available resources. However, for reference, we calculated our sensitivity in terms of the minimal effect size that we could detect with 80% probability, given our sample size (with two-tailed alpha = 0.05, using G*Power 3.1, https://www.psychologie.hhu.de/arbeitsgruppen/allgemeine-psychologie-und-arbeitspsychologie/gpower.html). As an effect size, we chose *f*^*2*^, e.g. for the change in *R*^*2*^ when adding the executive functioning predictor to a model with age and perceptual discrimination. With our sample of *N*=124 older adults (testing 1 of 3 regressors), we should have been able to detect effects as small as *f*^*2*^=0.065 with 80% power, i.e., somewhere between what Cohen (1988) called a small and a medium effect.

### 2.2 Recognition memory

#### 2.2.1 Materials

**Fig. 1:**
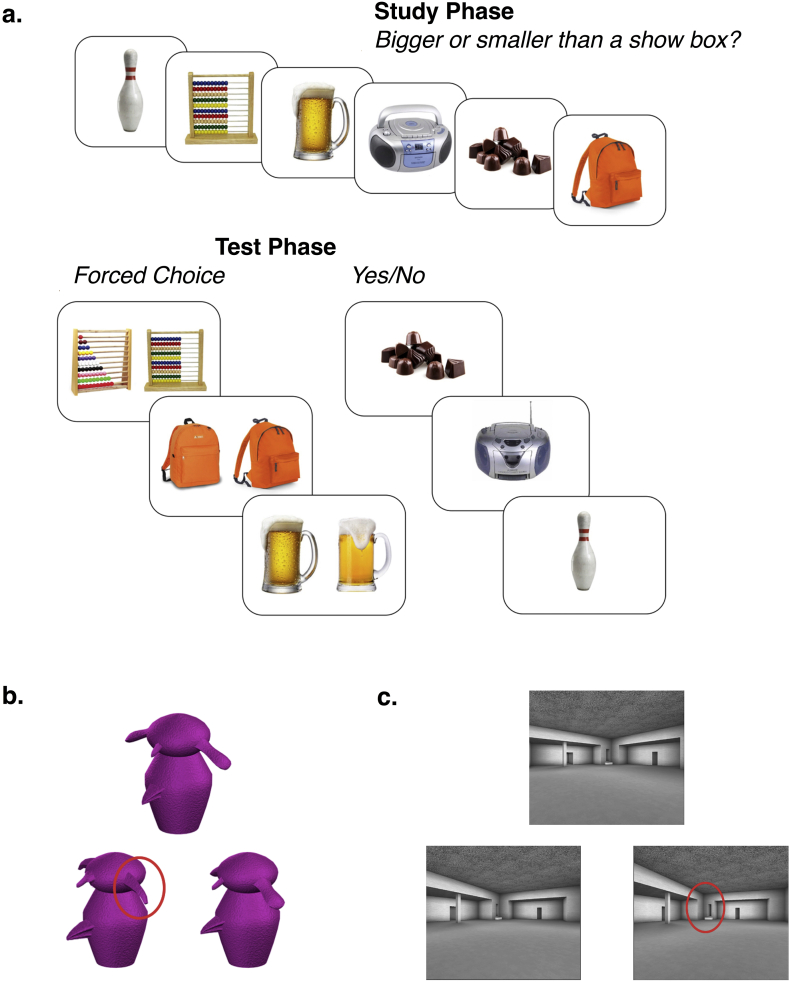
Schematic of the experimental paradigms. (a) Recognition memory task. During study, participants were shown objects and made a size judgment for each object (top). In the test phase (bottom), target and lure were either presented simultaneously (Forced Choice task), or either the target or the lure object was shown (Yes/No task). (b) Example of a trial of the high ambiguity object discrimination task. Red circles were not present in the actual task and are used here to illustrate which feature was different in the respective trial. (c) Example of a trial of the high ambiguity scene discrimination task. (For interpretation of the references to colour in this figure legend, the reader is referred to the web version of this article.)

The recognition memory task is summarized in Fig. 1a. Stimuli were 200 colour exemplar pairs of objects (a total of 400 images) obtained from online sources, including Google Image Search (Mountain View, CA) and publicly available stimulus sets (e.g., http://konklab.fas.harvard.edu). Each pair consisted of two images with high feature overlap that depicted the same type of object (e.g. “abacus”, “backpack”). Lures and targets could not be distinguished on the basis of conceptual representations or single features (shape, colour, pattern). Rather, discriminating exemplars relies on high fidelity representations of each object. Similarity ratings for exemplar pairs by an independent set of participants were used to create stimulus lists matched on average target-lure perceptual similarity. The assignment of lists to either task format (Forced Choice or Yes/No) was counterbalanced across participants.

#### 2.2.2 Procedure

After providing written informed consent, volunteers began with a practice block for the recognition memory tests with explicit instructions that a mix of targets and perceptually similar lures would be presented, and to endorse only exemplars that are perceptually identical to that which they had studied. To ensure task comprehension, participants were provided feedback on their performance during the practice block. Following successful completion of the practice task, participants moved on to complete two blocks of the study phase presenting 100 images per block for 3000 ms each. To direct attention to the features of the objects and ensure that all participants engaged in similar processing, participants were asked to make a Bigger/Smaller size judgment of the objects (‘*Is the object bigger/smaller than a shoe box*?’). Participants pressed the ‘z’ key for bigger and ‘m’ for smaller.

Before the test phase, participants were asked to count backwards from a three-digit number in steps of seven for 60 s. The order of Yes/No (YN) and Forced Choice (FC) test format was counterbalanced across participants. Each test format comprised 100 trials. For the FC format, target and lure of a stimulus pair were presented at the same time. Participants were asked to endorse either the stimulus on the left- (‘z’ key) or the right-hand (‘m’ key) side of the screen as old. In the YN format, either the target or the lure stimulus was shown in isolation and participants were asked if the item on the screen was old (i.e., identical to that which was studied), which they expressed with the ‘z’ key for ‘yes’ or ‘m’ key for ‘no’. Whether the target or the respective lure of a stimulus pair was shown was counterbalanced across participants.

#### 2.2.3 Scoring

We computed the discriminability index *d’* to quantify the separation between distributions for old and new items and allow comparison between YN and FC tasks (Bayley, Wixted, Hopkins, & Squire, 2008; Trelle et al., 2017). *d’* for FC and YN, respectively, was calculated as follows:

$$\mathrm{FC}\;d^{\prime }=\frac{1}{\sqrt{2}}\left(\mathrm{zHits}-\mathrm{zFalse Alarms}\right),$$

$$\mathrm{YN}\;d^{\prime }=z\left(\mathrm{Hits}\right)-z\left(\mathrm{False Alarms}\right).$$

In cases where the proportion would have been 0 or 1, we applied a correction to avoid a *d’* of infinity: 0% misses were recoded as 1/(2*N*)=0.005 and 100% hits as 1–1/(2*N*)=0.995 with *N* referring to the number of trials (MacMillan & Creelman, 1991). The signal-detection criterion in the Yes/No task was calculated as:  *YN c* =  − (*zHits* + *zFalse Alarms*)/2

### 2.3 Perceptual discrimination

Participants completed two perceptual discrimination (oddity) tasks, one for novel objects (greebles) and one for novel scenes.

#### 2.3.1 Materials

The perceptual discrimination tasks included computer-generated scene and object (greebles) stimuli, which have been used before to examine medial temporal lobe involvement in complex perceptual processing (objects from Barense, Gaffan, & Graham, 2007; scenes from Lee et al., 2005). The use of novel objects (greebles) and scenes meant that stimuli were unfamiliar to participants and did not carry semantic associations. Each stimulus display consisted of either three objects or three scenes (Fig. 1b, c). In the high ambiguity condition, stimuli were shown from different viewpoints and characterised by high feature overlap such that the same basic properties of the scenes or objects, respectively, were found in all exemplars (e.g., greeble body shape and position of appendages; configuration of columns and windows in scenes). In the low ambiguity condition, stimuli were mismatched on basic perceptual features, such that scenes and objects could be distinguised without taking into account conjunction of features and different viewpoints. The low ambiguity condition served as a control to measure basic perceptual discrimination performance and faciliate interpretation of performance in the high ambiguity condition.

#### 2.3.2 Procedure

Perceptual discrimination tasks were completed after the memory test. The order in which object and scene tasks were completed was counterbalanced across participants. To familiarise participants with the demand on feature conjunctions for the high ambiguity trials, participants completed ten practice trials during which the experimenter pointed out the differences between test exemplars if the participant answered incorrectly. A total of 60 trials were included in each task, 36 of which belonged to the high and 24 to the low ambiguity condition. During the main experiment, participants had 15 s to make a response with the keys ‘1’, ‘2’, or ‘3’ to indicate which of the three stimuli (top, bottom left, or bottom right, respectively) they believed to be different. Timed out trials were marked as incorrect.

Stimuli for recognition and discrimination tasks were presented in *MATLAB* (Mathworks, Inc., USA) in *Cogent 2000* (Cogent 2000 team at the FIL and the ICN and Cogent Graphics by John Romaya at the LON at the Wellcome Department of Imaging Neuroscience).

#### 2.3.3 Scoring

For group comparisons in high ambiguity perceptual discrimination we controlled for possible individual differences in basic perceptual discrimination ability (as indexed by the low ambiguity condition) by computing a standardised difference score *(z(% correct high ambiguity trials* - *% correct low ambiguity trials)*). For the regression analyses we aimed to capitalise on information from the two tasks by combining the two high ambiguity discrimination scores into a composite score as the mean of corrected object and scene values.

### 2.4 Executive function

#### 2.4.1 Materials and Procedure

Neuropsychological tests were carried out after memory and perception tests were completed. Following prior work (Hedden et al., 2012; Trelle et al., 2017), we used three tests to capture different aspects of executive functioning: 1) Working Memory, measured using Digit Span Forward and Backward (Wechsler Adult Intelligence Scale; Wechsler, 2008), 2) Attention and Mental Flexibility, measured using Trail making Tests A and B (D-KEFS; Delis, Kaplan, & Kramer, 2001), and 3) Verbal Fluency, measured using FAS letter fluency (D-KEFS; Delis et al., 2001). Each of these components are important for supporting strategic memory retrieval processes, which entails a) generating an appropriate search strategy (measured by verbal fluency), b) holding retrieved details in mind (measured by working memory), and c) flexibly changing one's strategies within the same task (measured by Trails B).

#### 2.4.2 Scoring

To index the multifaceted nature of executive functions (EF) as a proxy for strategic retrieval abilities, we created a composite score comprised of *z*-scores on Trails B (recoded such that higher values reflected better performance), Digit Span Total (forward + backward) and Verbal Fluency, based on their inter-correlations (Supplementary Material, section 1.a; |*r|* between 0.2 and 0.32; all *p*<.05). Similar strategies have previously been used by other studies (Craik, Eftekhari, Bialystok, & Anderson, 2018; Rhodes & Kelley, 2005; Trelle et al., 2017).

### 2.5 *Statistical analysis*

Statistical analysis was carried out using R Studio Version 1.2 (RStudio, Inc., 2013). The key analyses for this publication are shown in a corresponding R Markdown document that is available here as Supplementary Material (abbreviated as Suppl.). All summary data and the full length R Markdown script including all analyses conducted for this publication can be found on the Open Science Framework (https://osf.io/mcyh9/). All software packages used in our analysis are cited in the Markdown script (Suppl., section 8).

#### 2.5.1 Age-group differences in memory, perception and executive function

Age-group effects on neuropsychological test scores were assessed using *t*-tests with adjustments to the degrees of freedom where Levene's test revealed heterogeneity. We carried out a mixed ANOVA on memory *d*’ scores with the between-participants factor of Age Group and Task Format (FC, YN) as a repeated measure. No participants performed at chance level for the low ambiguity control condition (Suppl., 5.e.iii). We therefore did not exclude any participants on the basis of poor low-level perceptual abilities. Differences in the perceptual task were assessed with an independent samples *t*-test on corrected standardised discrimination scores (high-low ambiguity) for the PD composite as well as for objects and scenes*.* Removal of univariate and multivariate outliers from these analyses did not alter the results unless specifically reported in the results.

#### 2.5.2 Individual Differences Analyses

We used multiple regression to determine predictors of individual differences in FC and YN *d’* scores among older adults. We operationalised strategic retrieval as the composite in the executive functioning (EF) scores and representational quality as the composite of high ambiguity object and scene perceptual discrimination (PD), controlled for low ambiguity scores. We chose to use composites for both factors of interest to improve the stability of our estimate of each factor and reduce noise in the predictor variables. We further conducted supplementary analyses to show regression results for analyses with sub-scores of each composite

Predictors of interest were age, the perceptual discrimination composite and the executive functioning composite score. All models were also tested with sex and years of education as nuisance regressors. All predictors were converted to *z*-scores to obtain standardised *beta* regression coefficients. Model selection was done on the basis of complementary data-driven methods and a hypothesis-driven approach. Data-driven methods involved forward and backward stepwise regressions and best subset regressions, which determine the best set of predictors for different model sizes (one predictor to maximum number of predictors, in our case *n*=6). In the first step, data-driven approaches indicated that education and sex made no meaningful contribution to neither the Forced Choice nor Yes/No models (Suppl., 7.a-b.). Both variables were therefore excluded from further detailed model comparisons. In the second step we followed up on these results in more detail using hypothesis-driven analyses with predictors of interest being age, perceptual discrimination and executive functioning scores.

Finally, we tested whether executive functioning could explain additional variance in the Yes/No scores after controlling for performance on a familiarity-based memory test. This was done in two steps: 1) running a regression model on Yes/No scores using Forced Choice scores as a predictor and then 2) using the residuals from this model as dependent variable in a model with age, EF and PD as predictors.

#### 2.5.3 Model diagnostics

Details for model diagnostics regarding assumptions, outliers and influential cases can be found in Suppl., 7.e. Briefly, we tested whether assumptions of normality of residuals, homoscedasticity and absence of multicollinearity were met. After selection of the best fitting model for YN and FC *d’* scores, respectively, we tested whether the models were robust to the removal of outliers and influential cases. Influential cases were identified using a set of diagnostics calculated with the *influence.measures* function in the *R stats* package (Fox & Weisberg, 2018; R Core Team, 2019; metrics: centred leverage/hat values, Cook's distance, standardised residuals, DFBetas for each predictor, Mahalanobis Distance). Removal of all identified outliers and influential cases did not alter the regression results. Here we present the results from the full sample.

#### 2.5.4 Robustness analyses

We tested the robustness of our findings by 1) using the raw memory scores as the outcome variable (with the YN scores defined as (proportion of Hit trials+proportion of Correct Rejection trials)/2; and with proportion of correct trials for FC), 2) using either scene or object corrected scores as predictors instead of the PD composite, and 3) by using the individual subs-scores of the EF composite as predictors.

#### 2.5.5 Test of Differences in Slope: Linear mixed model

To confirm the differential contribution of perceptual discrimination and executive functioning to Forced Choice and Yes/No performance, respectively, we ran a linear mixed model across the *d’* scores of both tasks with random effect of participant and fixed effects of age, task format (FC or YN), type of cognitive process (EF or PD) and performance for the cognitive predictor as measured with z-scores (denoted *Z* Cognition). We also controlled for the interaction of age with task format and cognitive process. Thus, the model formula was as follows:

*d’=b*_*0*_*+ b*_*1*_ × *Age + b*_*2*_ × *Age* × *Format +b*_*3*_ × *Age* × *Z Cognition + b*_*4*_ × *Format* × *Cognitive* P*rocess* × *Z Cognition + (1|Participant ID).*

We were specifically interested in the three-way interaction between task format (FC, YN), type of cognitive process (EF, PD) and scores on the cognitive predictor, in order to test for differences in the slopes for the associations between the two memory tasks and the two cognitive predictors. Such an interaction would provide direct evidence for the possibility that EF and PD performance differentially influence performance in the YN and FC test formats, respectively.

Assumptions of linearity, normality of residuals and homoscedasticity were met. The results were robust to removal of influential cases and extreme standardised residuals (Suppl., 7.g.i.).

#### 2.5.6 Relative importance of predictors: Dominance analysis

Our exploratory correlation analyses revealed small to moderate correlations between all variables of interest (Forced Choice, Yes/No, PD, EF, age), suggesting that both processes contribute to performance. As noted above, the mixed model tested for differences in the relative contribution of perceptual discrimination and executive functioning to mnemonic discrimination as a function of task demands. We followed up these findings by determining for each task format separately the relative contribution of the EF and PD predictors. This analysis would inform the nature of the interaction of task format and cognitive predictor tested using the mixed model: if a statistically greater contribution of perceptual discrimination could be shown for FC and the same was true for executive functioning in the YN format, then this would suggest a complete dissociation. In contrast, if only one of these analyses produced significantly greater importance of one predictor over another, this would be an indicator of a partial interaction of cognitive process and task format. To test for such differences in the relative contributions of perceptual discrimination and executive functioning to the two task formats we conducted a dominance analysis implemented with the R package *dominanceanalysis* (Bustos Navarrete & Coutinho Soares, 2020).

Dominance analysis provides an intuitive understanding of the contribution of a predictor because it establishes relative importance based on changes in *R*^*2*^ as a function of adding predictors of interest to a model (Azen & Budescu, 2003; Nimon & Oswald, 2013). The method considers each pair of predictors (e.g. perceptual discrimination and executive functioning) and compares the change in *R*^*2*^ that can be attributed to a predictor as it is added to each possible subset of the model with 1 to *p*=maximum number of predictors (Budescu, 1993; Chevan & Sutherland, 1991). A predictor is said to have *complete dominance* over another when it contributes more variance to the outcome in *every* sub-model regardless of model size (Azen & Budescu, 2003). In our case this includes models with one, two or three predictors.

Dominance weights were estimated over 1000 bootstrap samples to provide a means for a statistical test of differences in the relative importance of predictors (Azen & Budescu, 2003). In each bootstrap sample a predictor is given a score of 1 when it explains more variance in the dependent variable than its competitor (on the basis of the *R*^*2*^ value calculated in a given bootstrap sample), and a value of 0 when the opposite is true. When neither predictor dominates the other, a value of 0.5 is given for the comparison. The mean dominance metric across bootstrap samples, the dominance weight, can then take on any value between 0 (predictor *A* is being dominated completely by predictor *B* in all samples) and 1 (predictor *A* dominates over *B* in all samples). If the mean dominance weight ± its standard error for a given pair of predictors does not include neutral 0.5 level, complete dominance could be established (for more methodological details see Suppl., 7.f.iii.).

## 3 Results

### 3.1 Age-group differences in memory, high-ambiguity perception and executive function

#### 3.1.1 Age deficits in Forced Choice and Yes/No tests are equivalent

**Fig. 2:**
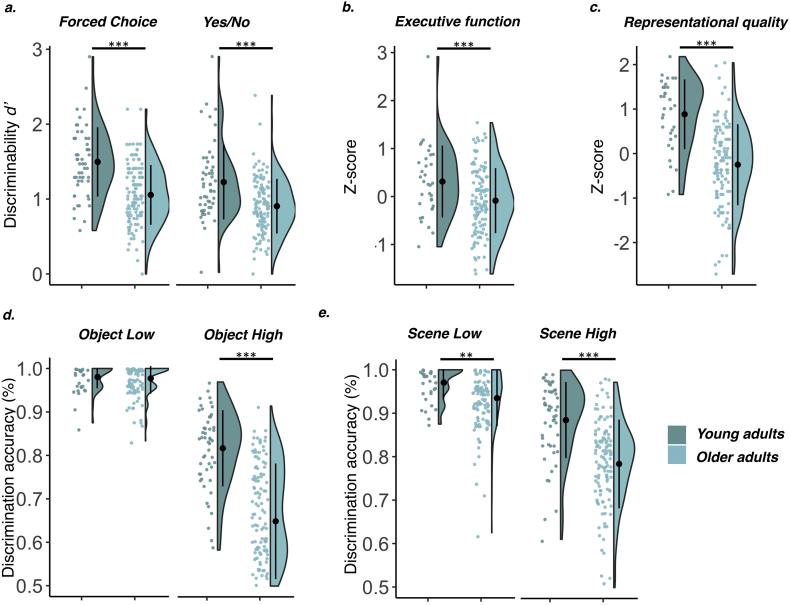
(a) Age group differences in Forced Choice and Yes/No *d’* scores, (b) executive functioning composite score, (c) perceptual discrimination composite score, (d) accuracy in object discrimination under low and high feature ambiguity, (e) accuracy in scene discrimination under low and high feature ambiguity. Note: One extreme outlier on the object high task in older adults is not shown in the plot. Outliers did not alter the significance of the effects shown here. ***p<.001, **p<.01.

We predicted that older adults would perform worse than younger adults in both tasks and that all participants would perform better in the Forced Choice test format than the Yes/No test format (Trelle et al., 2017). Results from the 2 × 2 mixed ANOVAs on recognition memory measures with between-participants factor Group (Young, Old) and within-participants factor Test Format (FC, YN) confirmed both hypotheses (although see Suppl. 5.d.iv., which suggests that task order influences the magnitude of the difference between FC and YN performance). There was a significant main effect of Group showing that younger adults had higher discriminability scores (*F*(1,170)=37.88, *p*<.001, *η*_*p*_^2^=0.18) and a main effect of Format confirming better performance for the FC task (*F*(1,170)=16.63, *p*<.001, *η*_*p*_^2^=0.09). There was no interaction (*F*<1), indicating that the difference in performance between Forced Choice and Yes/No was of similar magnitude across age groups. Use of proportion correct rather than *d*’ scores did not affect the results (Suppl., 5.c.). Group means are shown in Table 2 and Fig. 2.

**Table 2:** Memory and perception scores by age group.

	**Younger adults**	**Older adults**
**Memory scores**		
*Forced Choice*		
Correct	0.84 (0.07)	0.76 (0.08)
*Yes/No*		
Hits	0.80 (0.10)	0.83 (0.10)
False Alarms	0.35 (0.13)	0.54 (0.13)
Correct Rejections	0.65 (0.13)	0.46 (0.13)
Misses	0.20 (0.10)	0.17 (0.10)
**Perceptual discrimination accuracy**		
Object Low	0.98 (0.03)	0.98 (0.34)
Object High	0.82 (0.09)	0.65 (0.13)
Scene Low	0.97 (0.04)	0.94 (0.06)
Scene High	0.89 (0.09)	0.78 (10)
Discrimination composite (*z*)⁎	0.88 (0.78)	−0.25 (0.91)

⁎ Mean of corrected object and scene discrimination scores (High-Low ambiguity).

A mixed ANOVA with Group as between-participants factor (Young, Old) and Response Type (Hits, Correct Rejections; *F*(1,170)=54.42, *p*<.001, *η*_*p*_^2^=0.24) confirmed that age-related differences in the Yes/No test were driven by an increase in false alarms (*t*(99.47)=8.53, *p*<.001, *d*=1.40), whereas hit rate did not differ by age group (although a trend towards higher hit rates in older adults was observed: *t*(95.83)=−1.78, *p*=.079, *d*=0.30). Finally, older adults also had a more liberal response criterion than younger adults (*t*(171)=−6.25, *p*<.001, *d*=1.04).

#### 3.1.2 Age-related differences in high ambiguity perceptual discrimination

We predicted that older adults would be impaired on perceptual discrimination tasks when stimuli shared overlapping features. Independent samples *t*-test confirmed that older adults had significantly lower composite scores for high ambiguity discrimination controlled for lower-level perceptual processing (*t*(152)=6.59, *p*<.001, *d*=1.28). This age deficit was evident for discrimination of highly similar objects (*t*(79.63)=9.12, *p*<.001, *d*=1.41) and scenes (*t*(152)=3.17, *p*=.002, *d*=0.62). In other words, older adults experienced a larger decline in performance compared to younger adults when feature overlap was increased from low to high. Perceptual discrimination scores are shown in Table 2 and Fig. 2. Robustness analyses revealed that these effects were not affected by the 15 s time constraint (Suppl., 5.e.iv).

#### 3.1.3 Age-related differences in executive function

We tested for age deficits in the EF composite and its sub-scores. There was a significant difference between young and older adults on the executive functioning composite (*t*(154)=2.96, *p*=.004, *d*=0.57), which was driven by the Trails B score (*t*(154)=−9.48, *p*<.001, *d*=1.30). There was no age effect on Verbal Fluency or Digit Span Total scores (*t*(156)<1.5, *p*>.25).

### 3.2 Individual differences in false recognition in older adults

**Fig. 3:**
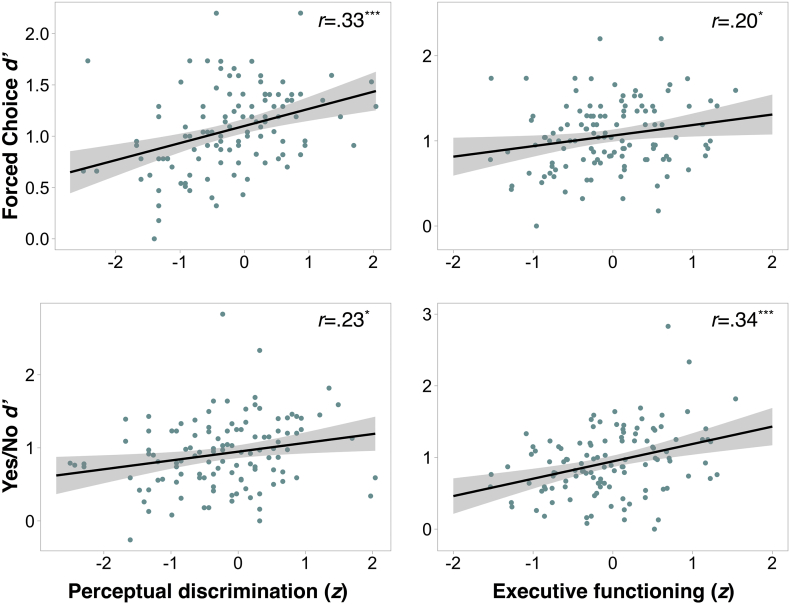
Correlations between variables of interest (Yes/No *d’*, Forced Choice *d’*, Perceptual Discrimination composite score, Executive Functioning composite score). Corrections for multiple comparisons are based on the Holm method.* *p*<.05, ** *p*<.01, *** *p* <.001.

We used multiple regression to examine the relationship between perceptual discrimination, executive function, and individual differences in memory performance on the FC and YN tasks among older adults. The *d’* scores for each memory task were the dependent variables of interest. The predictors of interest were age, the perceptual discrimination composite score and the executive functioning composite. Correlations between memory, perceptual and executive functions are shown in Fig. 3.

#### 3.2.1 Perceptual discrimination explains individual differences in Forced Choice performance

We predicted that representational quality, measured using a composite of high ambiguity object and scene discrimination, would be the primary factor explaining individual differences in Forced Choice performance in older adults. Consistent with this prediction, model comparison (Table 3) revealed that the addition of PD significantly improved model fit relative to a model with age alone (*F*(1,110)= 5.15, *p*=.016), whereas the addition of EF did not (*F*(1, 109)= 2.10, *p*=.121). Thus, the model that best describes Forced Choice performance is as follows: *FC* *d*’=2.28–0.02*Age+0.11*Perceptual *Discrimination.*

**Table 3:** Model coefficients for FC *d’*.

**Factor**	**Model 0**			**Model 1**			**Model 2**			**Model 3**		
	*F*(*df*)		*R*^*2*^_*adj*_	*F*(*df*)		*R*^*2*^_*adj*_	*F*(*df*)		*R*^*2*^_*adj*_	*F*(*df*)		*R*^*2*^_*adj*_
	13.49 (1111)		0.10	10.04 (2110)		0.14	8.02 (2110)		0.11	7.05 (3109)		0.14
	*β*	*SE*	*p*	*β*	*SE*	*p*	*β*	*SE*	*p*	*β*	*SE*	*p*
Age	−0.33	0.09	<0.001	−0.23	0.10	0.017	−0.31	0.09	<0.001	−0.23	0.10	0.019
PD				0.24	0.10	0.016				0.21	0.10	0.034
EF							0.14	0.09	0.127	0.09	0.10	0.306

*Note:* All models were significant at *p*<.001. *SE*=standard error of the mean. EF: executive functioning composite; PD: perceptual discrimination composite. ^⁎^*p*<.05, ^⁎⁎^*p*<.01, ^⁎⁎⁎^*p*<.001.

##### 3.2.1.1 Robustness analyses

When scores on the individual discrimination tasks were used in the FC model together with age, both object and scene corrected scores were significant predictors of Forced Choice *d’* scores (object high corrected: *β*=0.214, *SE*=0.096, *p*=.027; scene high corrected: *β*=0.182, *SE*=0.091, *p*=.049; Suppl., 7.d.i). Using the proportion of correct trials as the outcome measure also resulted in a significant effect of perceptual discrimination (*F*(2,110)=11.78, *p*<.001, *R*_*adj*_^2^=0.16; PD: *β*=0.266, *SE*=0.095, *p*=.006; Suppl., 7.d.ii).

#### 3.2.2 Executive functions explain individual differences in Yes/No performance

We predicted that executive function composite score, a proxy for strategic retrieval ability (see Methods 2.4.1), would be the primary factor explaining individual differences in Yes/No performance in older adults. Consistent with this prediction, model comparison (Table 4) revealed that the addition of EF significantly improved model fit relative to a model with age alone (*F*(1,110)=9.67, *p*=.002), whereas the addition of PD did not (*F*<2, *p*>.2). Thus, the model that best explains Yes/No performance is described by the regression equation: *YN* *d’*=2.68–0.02*Age+0.20*Executive Function.

**Table 4:** Coefficients for all models for YN *d’* in older adults.

**Factor**	**Model 0**			**Model 1**			**Model 2**			**Model 3**		
	*F*(*df*)		*R*^*2*^_*adj*_	*F*(*df*)		*R*^*2*^_*adj*_	*F*(*df*)		*R*^*2*^_*adj*_	*F*(*df*)		*R*^*2*^_*adj*_
	13.52 (1111)		0.10	7.42 (2110)		0.10	12.16 (2110)		0.17	8.12 (3109)		0.16
	*β*	*SE*	*p*	*β*	*SE*	*p*	*β*	*SE*	*p*	*β*	*SE*	*p*
Age	−0.33	0.09	<0.001	−0.28	0.10	0.005	−0.29	0.09	<0.001	−0.27	0.10	0.006
PD				0.11	0.10	0.258				0.05	0.10	0.639
EF							0.27	0.09	0.002	0.26	0.09	0.004

*Note:* All models were significant at *p*<.001. *SE*=standard error of the mean. EF: executive functioning composite; PD: perceptual discrimination composite. ^⁎^*p*<.05, ^⁎⁎^*p*<.01, ^⁎⁎⁎^*p*<.001.

We also controlled for the influence of FC scores on YN to account for familiarity-based memory performance. Indeed, the effect of executive functioning remained significant in a model with age, EF and PD as regressors (*F*(3,109)=3.62, *p*=.015, *R*_*adj*_^2^=0.07; EF: *β*=0.212, *SE*=0.080, *p*=.009; Suppl., 7.d.iv).

##### 3.2.2.1 Robustness analyses

When the proportion of correct trials was used as the outcome variable, the effects of age and EF were the same as for *d’* (*F*(2,110)=13.30, *p*<.001, *R*_*adj*_^2^=0.18; EF: *β*=0.292, *SE*=0.087, *p*<.001; Suppl., 7.d.iii). The finding that executive functioning explained much of the variance in Yes/No performance could also be reproduced when using only Trails B or Digit Span Total but not Verbal Fluency as predictor (Suppl., 7.d.v). Controlling for task order in multiple regressions did not alter the contribution of age and executive functioning (Suppl., 7.d.vi). Next, we ran regression models for hits and correct rejections separately. Only the model for correct rejections but not that for hits showed an effect of executive functioning on individual differences in Yes/No performance (Suppl., 7.d.vii; *F*(3,111)=6.54, *p*<.001, *R*_*adj*_^2^=0.13). Finally, response criterion was unrelated to executive functioning (Suppl., 7.d.iix).

#### 3.2.3 Perceptual discrimination and executive function differentially contribute to Forced Choice and Yes/No Performance

**Fig. 4:**
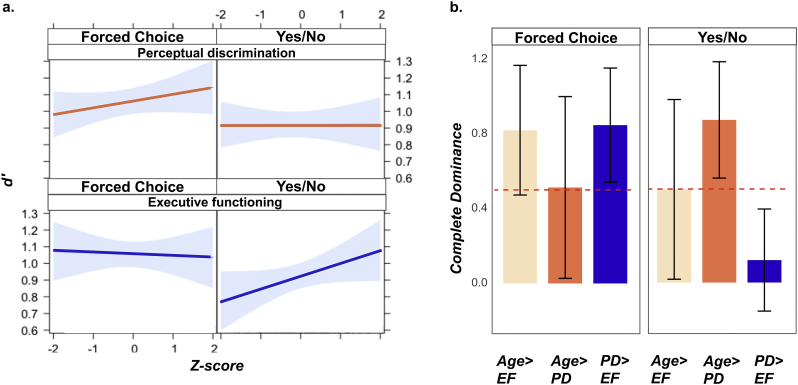
Differential importance of predictors. a. Results of the linear mixed model for *d’* memory scores. The plot shows the three-way interaction effect of task format (FC, YN), cognitive process (EF, PD) and performance on the cognitive predictors (z-scores). The plots shows an attenuation in the associations between perceptual discrimination and Yes/No performance and the association between executive functioning and Forced Choice performance, respectively, after controlling for all other effects in the mixed linear model as opposed to bivariate correlations with no covariates (see Fig. 3). b. Complete dominance metric for predictors used in the Forced Choice and Yes/No *d’* regression models. The y-axis represents the mean complete dominance metric across all bootstrap samples. EF: executive functions; PD: perceptual discrimination.

We used a linear mixed model to formally test differential contributions of PD and EF to FC and YN performance, respectively. The results confirmed the presence of a three-way interaction between task format, type of cognitive predictor and performance on the respective cognitive predictor (*β*=−0.256, *SE*=0.106, *p*=.016), even after controlling for age. Fig. 4a shows the associations between PD and EF with performance in the two task formats after adjusting for the influence of all other predictors in the model. The relationship between perceptual discrimination and Forced Choice remained apparent even after this adjustment. The opposite was true for Yes/No performance, where executive functioning scores contributed more to performance.

#### 3.2.4 Perceptual discrimination and executive function dominate Forced Choice and Yes/No performance, respectively

To establish dominance between predictors, the contribution of each predictor towards explaining variance in the dependent variable was compared across all possible model subsets containing any combination of age, perceptual discrimination and/or executive function. Within our sample, the PD regressor had complete dominance over the EF predictor in the Forced Choice task. This pattern of complete dominance of PD vs. EF was reproduced in 77% of bootstrap samples. As shown in Fig. 4b, the standard errors of the comparison of PD>EQ was above the 0.5 neutral line (*M*=0.85, *SD*=0.30), indicating that the PD predictor had complete dominance over EF.

For the Yes/No model, the analysis established complete dominance of executive functioning over perceptual discrimination in our sample, which was reproduced by 82% of bootstrap samples. Moreover, Fig. 4b shows that the standard error of the mean complete dominance metric for the comparison of PD>EF was well below the 0.5 line (*M*=0.12, *SEM*=0.27). These findings suggest that complete dominance of EF over PD in predicting Yes/No performance is generalisable beyond our sample. That is, in all possible subsets of Yes/No models, executive functioning contributed more to the proportion of variance explained than did the perceptual discrimination composite scores. The dominance analysis therefore informs the three-way interaction found in the linear mixed model by demonstrating a complete dissociation of the relative importance of perceptual discrimination and executive functioning to Forced Choice and Yes/No performance, respectively. In each task format, one of these two cognitive predictors is more important than the other in explaining individual differences in mnemonic discrimination.

## 4 Discussion

The current study investigated the contribution of individual differences in perceptual discrimination and executive functions to mnemonic discrimination of perceptually similar objects in cognitively unimpaired older adults. Moreover, it revealed how these two factors impact performance depending on key experimental design parameter, namely recognition memory test format. These investigations yielded three key findings. First, we replicate previous observations of age-group differences in Yes/No and Forced Choice recognition memory tasks with high target-lure similarity (Trelle et al., 2017) and in perceptual discrimination tests under conditions of high feature overlap (Ryan et al., 2012). We extend these findings providing evidence for effects of chronological age on both memory and high ambiguity perception within the older group. Second, using both data-driven and hypothesis-driven individual differences analyses, we provide strong evidence for contributions of high ambiguity perceptual discrimination and executive function to individual differences in mnemonic discrimination in a large cohort of older adults. Finally, we demonstrate that the relative importance of each factor differs as a function of test format, such that perceptual discrimination best explains Forced Choice performance, whereas executive function best explains Yes/No performance. Collectively, these results significantly extend our understanding of the factors that contribute to individual differences in mnemonic discrimination among older adults. These results are critical given the potential utility of mnemonic discrimination tasks in clinical contexts.

Our finding of age-related deficits in the Yes/No task is in line with a plethora of other studies demonstrating significant impairments in recollection and mnemonic discrimination in older adults (Cohn et al., 2008; Koen & Yonelinas, 2014; Reagh et al., 2016, Reagh et al., 2018; Stark & Stark, 2017; Trelle et al., 2017; Yassa et al., 2011). Here we demonstrate that although both perceptual discrimination and executive function were correlated with Yes/No performance, the relationship between executive functioning and memory was significantly stronger for the Yes/No compared to the Forced Choice task. Moreover, when executive functions are taken into consideration, perceptual discrimination performance does not provide further information to explain individual differences in Yes/No performance. While sufficient representational quality is undoubtedly a prerequisite for the availability of item details that can be diagnostic for recognition, they were insufficient to explain deficits in the YN task. This is consistent with behavioural and lesion studies which have shown that frontally-mediated cognitive control is essential for high performance on tasks that require recall as opposed to traditional recognition (Jacoby, 1991; Johnson, O'Connor, & Cantor, 1997; Parkin, Yeomans, & Bindschaedler, 1994; Yonelinas, 2002). In the context of this task, older adults, and those with poor executive function in particular, may have had difficulty implementing effective retrieval strategies, such as recall-to-reject, which is known to place significant demands on cognitive control (including working memory maintenance and evaluation processes), and which is critical for minimising false alarms to lures (Cohn et al., 2008; Gallo et al., 2006; Migo et al., 2009; Trelle et al., 2017). This possibility is in line with the observation that age-related reductions in target-lure discriminability in our Yes/No task were driven by an increase in false alarms for older adults. They also dovetail with our finding of a specific relationship between executive functioning and individual differences in correct rejections but not hits in the Yes/No task among older adults. Importantly, response bias was unrelated to executive functioning. These data strongly support the notion that executive functioning is particularly crucial in cases where older adults have to use a recall-to-reject strategy. It may be surprising then, that we do not find a greater age deficit in the Yes/No as opposed to the Forced Choice task. This finding reproduces results from Trelle et al. (2017). Despite the relatively greater reliance on cognitive control in the Yes/No task, performance differences in the two task formats within participants was only marginally related to executive functioning (Suppl. 7.d.ix), which may explain the absence of an age by task format interaction.

The finding that Yes/No performance was related to executive function is also compatible with prior work demonstrating that individual differences in delayed recall scores explain variance in Yes/No mnemonic discrimination in older adults ((Bennett et al., 2019); Migo et al., 2014; Stark, Yassa, & Stark, 2010; Toner et al., 2009). Importantly, neuropsychological tests of delayed recall (e.g., word list recall) are known to rely on both hippocampal-dependent pattern completion and PFC-dependent strategic retrieval processes (Chang et al., 2010; Dolan & Fletcher, 1997). The observed effect of executive function on performance in the present study may index PFC-mediated aspects of recall that likely also contribute to that relationship, in addition to hippocampal-mediated effects. Consistent with this idea, relationships between memory performance and executive function have also been observed using different memory paradigms that place similar demands on strategic retrieval processes (Davidson & Glisky, 2002; Perrotin, Tournelle, & Isingrini, 2008). Although the precise set of neuropsychological tasks used to index executive function differs across studies, the composite scores tend to commonly survey inhibition, working memory, strategic search and mental flexibility (de Faria, Alves, & Charchat-Fichman, 2015), which are all processes that support the controlled search for and evaluation of item details during post-retrieval monitoring (Rhodes & Kelley, 2005).

It should be noted, however, that evidence for the role of executive functions in mnemonic discrimination is mixed, with some studies failing to identify effects (Davidson et al., 2019; Toner et al., 2009), and others suggesting that executive function impacts performance (Camfield, Fontana, Wesnes, Mills, & Croft, 2018; Trelle et al., 2017). One difference across studies is the use of an old/similar/new response option and inclusion of novel foils in prior work, as compared to the old/new response option with targets and similar lures in the current study. Although existing evidence has demonstrated that age-related deficits in lure discrimination performance remain consistent across response formats and many other task modifications, such as task instructions, (Stark et al., 2015), we cannot rule out the possibility that this difference in task format contributed to differences across studies with respect to effects of executive function on performance. Another key difference across studies is that of sample size, with null findings largely coming from studies with smaller samples (*N* = 20–38; Davidson et al., 2019; Toner et al., 2009). Indeed, our study includes one of the largest samples in the literature on individual differences in mnemonic discrimination, thereby likely increasing the ability to detect relationships between executive function and memory performance. Although future work is needed to examine effects of response options and task instructions on these relationships, the present findings suggest that executive function can significantly impact mnemonic discrimination. Future work aimed at isolating MTL-dependent processes using mnemonic discrimination tasks might benefit from minimising strategic retrieval demands, or accounting for variance related to executive function within the target population.

Our results also suggest that mnemonic discrimination deficits in older adults arise not only due to factors associated with strategic retrieval demands. Specifically, we demonstrate age-related decline in Forced Choice performance (replicating work by Trelle et al., 2017), a test format that is known to minimise demands on hippocampal-mediated retrieval (e.g. pattern completion; Holdstock et al., 2002; Migo et al., 2009) including strategies such as recall-to-reject (Guerin et al., 2012; Migo et al., 2009; Trelle et al., 2017). Consistent with this idea, executive function was not a significant predictor of FC performance, whereas perceptual discrimination of stimuli with overlapping features explained significant variance. These results complement and extend our prior work examining group differences in high ambiguity perception in relation to Forced Choice mnemonic discrimination (Trelle et al., 2017), but here using a different perceptual discrimination measure that is also known to rely critically on the MTL (Barense et al., 2010; Lee et al., 2005).

To our knowledge, this study is the first to establish a continuous relationship between individual differences in high ambiguity perceptual discrimination and mnemonic discrimination performance in healthy older adults. We interpret this relationship in terms of common representational demands across perceptual and mnemonic tasks that require disambiguating stimuli with overlapping features, such that poorer perceptual discrimination performance indexes reduced availability of complex, conjunctive stimulus representations supported by the MTL (Barense et al., 2007; Bussey & Saksida, 2005; Kent et al., 2016). Such representations should be critical for performance across both Forced Choice and Yes/No format. Indeed, perceptual discrimination scores correlated with performance across test formats, but this relationship was significantly stronger in the Forced Choice model. In contrast, perceptual discrimination scores were not retained in the final Yes/No model when accounting for executive functioning. Our results suggest that while the availability of complex perceptual representations is important for minimising false recognition, the degree to which this factor is the key driver of memory performance depends on the extent to which the task incurs additional cognitive demands, such as demands on controlled retrieval processes.

These data also add to a growing body of evidence demonstrating that perceptual discrimination is impacted with age under conditions of high feature ambiguity (Burke et al., 2012; Ryan et al., 2012), and further support the idea that age-related declines in perceptual processing are critically linked to age-related declines in episodic memory, perhaps because these processes rely on common representations formed by the MTL (Barense et al., 2007; Bussey & Saksida, 2005; Kent et al., 2016). Older adults with coarser representations likely have poorer pattern separation (Berron et al., 2016; Kent et al., 2016; Yassa, Mattfeld, Stark, & Stark, 2011; Yassa & Stark, 2011) and must rely more on single features or simple feature conjunctions to distinguish stimuli (Burke et al., 2012; Newsome et al., 2012; Reagh & Yassa, 2014; Ryan et al., 2012). The result is an increase in feature interference between previously viewed and novel stimuli with overlapping features (Barense et al., 2012; Johnson et al., 2016), impairing discrimination accuracy, both in the context of perceptual tasks and memory tasks. Consistent with this interpretation, prior work from our lab (Trelle et al., 2017) and others (Newsome et al., 2012) have demonstrated that reducing feature-level interference can improve Forced Choice mnemonic discrimination and perceptual discrimination accuracy in older adults.

Although we interpret the present results primarily in terms of representational quality (i.e., ability to resolve interference between objects with overlapping features), and executive function (i.e. ability to engage processes that support maintenance and evaluation of these representations), they can also be interpreted in terms of differential contributions of familiarity and recollection processes to performance across test formats. Specifically, prior work suggests that Forced Choice performance, when allowing for direct comparison of targets and corresponding lure, can be supported by cortical familiarity signals, whereas a Yes/No test requires hippocampal-dependent recollection (Holdstock et al., 2002; Migo et al., 2009; Norman, 2010; Trelle et al., 2017). As we did not collect data regarding response strategies during retrieval, we cannot draw strong conclusions regarding the strategies employed across test formats in the present study. Importantly, our results suggest that the success of a strength-based familiarity signal may be linked to the availability of stimulus representations that can effectively disambiguate perceptually similar targets and lures. This may explain why the presence of age-related differences in tasks that can be supported by stimulus familiarity often depends on target-lure similarity (Bastin & Van der Linden, 2003; Koen & Yonelinas, 2014; Trelle et al., 2017; Yonelinas, 2002).

Regardless of the framework chosen, it should be noted that Forced Choice and Yes/No tasks are unlikely to be “process pure”, meaning that scores on both tests almost certainly involve a combination of trials where item details were recollected and those where items were recognised without recall of specific features. Nevertheless, we argue that the difference in task demands renders our contrast of FC and YN formats capable of teasing apart age-related decline in the availability of stimulus representations that can support discrimination performance, versus the ability to effectively access and utilise these representations to support performance. This is predicated on the findings of prior studies, which demonstrated that the provision of retrieval support in a Forced Choice task enhances discrimination performance, at least in part by increasing the accessibility of stored representations (Guerin et al., 2012; Migo et al., 2009), and is consistent with the significant increase in performance observed across age groups in the Forced Choice test relative to the Yes/No test in the present study and in past work (Trelle et al., 2017). Most importantly, our interpretation of representational quality as a key factor predicting Forced Choice performance due to the provision of retrieval support holds whether participants relied on a strength-based familiarity signal or were simply able to recall item details less effortfully without the need to engage strategic retrieval processes.

It is possible that the perceptual discrimination measure used here is also not process-pure, and may be influenced by other factors. For example, lower-level perceptual processes (such as those on the level of the retina or earlier visual cortical regions) could contribute to impairments in more complex visual discrimination tasks. Behavioural evidence suggests an association between low-level perceptual processes, such as visual acuity and contrast sensitivity, and the accuracy of mnemonic discrimination of highly similar targets and lures (Davidson et al., 2019). Neural evidence further points to age-related dedifferentiation in earlier visual regions that feed into the MTL (Bowman, Chamberlain, & Dennis, 2019; Carp, Park, Polk, & Park, 2011; McDonough, Cervantes, Gray, & Gallo, 2014; Payer et al., 2006; Trelle, Henson, & Simons, 2019). Keeping in mind these potential contributions of low level perceptual processes to our representational quality regressor, we aimed to minimise their influence by controlling for low ambiguity discrimination performance on the high ambiguity discrimination measure (e.g., Object High – Low). Although the range in performance in the low ambiguity condition was small, demonstrating that no gross perceptual impairments were present within the current sample, the use of this difference score helped to account for small variation in basic perceptual processing within the sample. Thus, it is unlikely that deficits in basic perceptual processing were the main cause of the age effect on perceptual discrimination in the present data. Together with past work, the present findings underscore the relevance of perceptual processing on mnemonic processing in cognitive aging.

It is possible that perceptual discrimination deficits in older adults were due to deficits in executive functioning given the working memory component of oddity tasks. Critically, the present study measured both of these factors, providing an opportunity to explore this question directly. We did indeed observe a significant correlation between executive function and perceptual discrimination in the present sample. Importantly, however, we were able to include both measures in our regression analyses. If observed effects of perceptual discrimination were driven by executive functions, EF should have emerged as the primary predictor of Forced Choice performance. In contrast, both the model selection procedure and the dominance analysis showed that PD, but not EF was chosen as the defining predictor for Forced Choice performance. Together, the present results highlight the relationship between perceptual and mnemonic discrimination in the Forced Choice test format, but also indicate that executive functioning impacts many cognitive domains and is an important factor to consider in cognitive aging research.

In summary, our study provides novel insights into the cognitive factors that explain individual differences in mnemonic discrimination ability in healthy older adults. Notably, the present study focused on relatively less explored factors outside the realm of MTL-based mnemonic functions by also considering the perceptual processes supported by the MTL and by taking into account extra-hippocampal retrieval processes. These findings highlight the importance of both representational quality and strategic retrieval in mnemonic discrimination in healthy aging. That is, older adults do not only struggle with controlled retrieval of memory content, but they also face the challenge of operating on less distinctive stimulus representations. Our large sample allowed us to examine the relative contribution of these two factors by contrasting test formats with differential demands on strategic retrieval. Both hypothesis- and data-driven methods clearly converged to support a significantly greater role of perceptual discrimination abilities in explaining Forced Choice, and a greater role of executive function in explaining Yes/No performance.

These findings provide compelling evidence that multiple factors contribute to age-related decline in mnemonic discrimination, and that their influence depends on key experimental design decisions such as test format. They also highlight the presence of considerable variability in both memory and perceptual discrimination performance, even within a sample of cognitively unimpaired older adults. That is, although older adults perform worse on average than younger adults, there is significant overlap in the distributions of performance across groups. Understanding what drives this variability is critical, not only with respect to better characterising cognitive aging, but also in light of evidence that impaired performance on these types of tasks could signal increased risk of underlying preclinical AD pathology (Berron et al., 2019; Rentz et al., 2013; Webb et al., 2020). While additional work is needed to explore the neuroanatomical basis of differences across test formats, the present findings represent important initial steps towards developing a deeper understanding of individual differences in mnemonic discrimination in older adults.

## Author note

This research was supported by the 10.13039/501100000268BBSRC [grant number BB/L02263X/1], and was carried out within the 10.13039/501100000735University of Cambridge Behavioural and Clinical Neuroscience Institute, funded by a joint award from the 10.13039/501100000265Medical Research Council and the 10.13039/100010269Wellcome Trust. H.M.G. was supported by a 10.13039/501100000265Medical Research Council scholarship, R.N.H. by 10.13039/501100000265Medical Research Council programme grant [grant number SUAG/046 G101400], and J.S.S. by 10.13039/100000913James S. McDonnell Foundation Scholar award [grant number #220020333]. The funders had no role in the conceptualisation, analysis or publication of this data. The study was conducted at the Behavioural and Clinical Neuroscience Institute (BCNI) and the MRC Cognition and Brain Sciences Unit (CBU) at the University of Cambridge.

The authors would like to thank the funders for their support of their research. Special thanks go to Priyanga Jeyarathnarajah, Sarah Fox and Megan Thomson for help with data collection, to Mark Haggard for advice on data analysis, and to Morgan Barense for sharing task stimuli.

Preliminary results of this study have been presented at the following conferences: Aging of Memory Functions, 2018, Bordeaux, France; Cognitive Neuroscience Society Annual Meeting, 2019, San Francisco, United States; Cambridge Memory Meeting, 2020, Cambridge, United Kingdom.

Author credits – Gellersen et al., 2020.

## Declaration of Competing Interest

none.
